# Everything in Moderation - Dietary Diversity and Quality, Central Obesity and Risk of Diabetes

**DOI:** 10.1371/journal.pone.0141341

**Published:** 2015-10-30

**Authors:** Marcia C. de Oliveira Otto, Nikhil S. Padhye, Alain G. Bertoni, David R. Jacobs, Dariush Mozaffarian

**Affiliations:** 1 Division of Epidemiology, Human Genetics and Environmental Sciences, The University of Texas Health Science Center, School of Public Health, Houston, Texas, United States of America; 2 Center for Nursing Research, The University of Texas Health Science Center, School of Nursing, Houston, Texas, United States of America; 3 Department of Epidemiology & Prevention, Division of Public Health Sciences, Wake Forest School of Medicine, Winston-Salem, North Carolina, United States of America; 4 Division of Epidemiology & Community Health, School of Public Health, University of Minnesota, Minneapolis, Minnesota, United States of America; 5 Friedman School of Nutrition Science & Policy, Tufts University, Boston, Massachusetts, United States of America; Universitat de Lleida-IRBLLEIDA, SPAIN

## Abstract

Diet guidelines recommend increasing dietary diversity. Yet, metrics for dietary diversity have neither been well-defined nor evaluated for impact on metabolic health. Also, whether diversity has effects independent of diet quality is unknown. We characterized and evaluated associations of diet diversity and quality with abdominal obesity and type II diabetes (T2D) in the Multi-Ethnic Study of Atherosclerosis. At baseline (2000–02), diet was assessed among 5,160 Whites, Hispanic, Blacks, and Chinese age 45–84 y and free of T2D, using a validated questionnaire. Three different aspects of diet diversity were characterized including count (number of different food items eaten more than once/week, a broad measure of diversity), evenness (Berry index, a measure of the spread of the diversity), and dissimilarity (Jaccard distance, a measure of the diversity of the attributes of the foods consumed). Diet quality was characterized using aHEI, DASH, and a priori pattern. Count and evenness were weakly positively correlated with diet quality (r with AHEI: 0.20, 0.04), while dissimilarity was moderately inversely correlated (r = -0.34). In multivariate models, neither count nor evenness was associated with change in waist circumference (WC) or incident T2D. Greater food dissimilarity was associated with higher gain in WC (p-trend<0.01), with 120% higher gain in participants in the highest quintile of dissimilarity scores. Diet diversity was not associated with incident T2D. Also, none of the diversity metrics were associated with change in WC or incident T2D when restricted to only healthier or less healthy foods. Higher diet quality was associated with lower risk of T2D. Our findings provide little evidence for benefits of diet diversity for either abdominal obesity or diabetes. Greater dissimilarity among foods was actually associated with gain in WC. These results do not support the notion that “eating everything in moderation” leads to greater diet quality or better metabolic health.

## Introduction

“Eat everything in moderation”, or diet diversity, is a long standing public health recommendation. Yet, remarkably, dietary diversity has never been clearly defined nor measured, and thus little is known about its actual impact on human health. For example, it is unknown whether dietary diversity reduces intakes of unhealthy food components such as refined grains, processed meat, salt, or trans-fat, or–most relevantly–reduces risk of diet-related chronic diseases[1–5]. In addition, most [6–10], but not all [11] evidence to date linking dietary diversity and quality to weight gain and metabolic disorders including type II diabetes (T2D) incidence and mortality is limited to White populations.

Few studies have evaluated the role of dietary diversity on metabolic outcomes. A recent study showed a prospective inverse association between dietary diversity of fruit and vegetable intake and risk of T2D, which was independent of the amount of fruit and vegetable consumed [12]. A prior study evaluating the role of dietary diversity on body fat showed inverse association between diversity of vegetables intake and body fat; however, greater diversity in intakes of unhealthy foods such as bakery desserts, salty snacks and carbohydrates was associated with body fat [13]. These results suggest that dietary diversity may be particularly relevant to obesity and T2D, and that there may be potential differences in associations of dietary diversity for more healthy vs. less healthy foods.

One of the key barriers to assessment of dietary diversity has been the lack of standardized or appropriate methods to characterize it. In most prior studies, dietary diversity has been assessed by summing the number of different foods consumed in a given period of time (1–7 days), mostly across selected food groups of interest, such as fruits, vegetables, meat and dairy. These measures do not account for other relevant components of diversity such as the evenness of the distribution of foods across different food groups. Used in recent studies [14–16], the healthy food diversity index is a metric that reflects both evenness of the food distribution and diet quality, making it difficult to evaluate independent associations of this aspect of food diversity with metabolic outcomes. In addition, prior dietary measures do not allow investigation of similarities and differences between food items or the potential differences in associations of dietary diversity for more healthy vs. less healthy foods. To address these key gaps in knowledge, we systematically characterized novel metrics of dietary diversity and evaluated their associations with gain in waist circumference and risk of T2D in the Multi-Ethnic Study of Atherosclerosis (MESA).

## Methods

### Study Design

The MESA is a prospective cohort study designed to investigate risk factors associated with subclinical cardiovascular disease across race/ethnicities. Detailed information on study design and methods has been previously described [17]. Briefly, 6,814 participants free of clinical CVD were recruited in 2000 at six U.S. study centers (38% Whites, 28% Blacks, 22% Hispanics and 12% Chinese Americans), ranging from 45 to 84 years of age [17]. The present study was conducted according to the guidelines laid down in the Declaration of Helsinki and all procedures involving human subjects and patients were approved by the affiliated institutional review boards affiliated with each of the participating academic centers (Columbia University, Johns Hopkins University, Northwestern University, University of California, Los Angeles, University of Minnesota, Wake Forest University). All participants gave written informed consent. At baseline, participants reported usual dietary intake over the previous year using a validated 120-item food frequency questionnaire (FFQ), with modifications to include Chinese foods and beverages (23, 24).

#### Dietary assessment

Usual food intake over the previous year was assessed at baseline examination using a Block-type 120-item self-administered food frequency questionnaire (FFQ) [18], modified to include items from the Chinese cuisine [19]. Participants reported frequency of food consumption (categories of times per day) and portion sizes (small, medium and large) for selected foods and beverages. Criterion validity of the modified MESA-specific FFQ was evaluated against plasma lipid concentrations within the MESA cohort [20]. The current analyses were performed using the most up-to-date MESA dietary dataset, i.e. after quality control measures were taken to minimize underreporting in 19 food items (apples, orange and other juice, lettuce, spinach, potato, dark bread, crackers, chips, cheddar and cottage cheese, yogurt, hamburger, ham, chili, sausage, chocolate and white doughnuts), including re-scanning of original questionnaires. In a subset of participants missing original FFQs, missing information on food frequency and serving sizes of 19 FFQ items was imputed using multinomial regression or ordinal regression models. Nutrient intake was estimated for each FFQ item using the Nutrition Data System for Research (NDS-R database; Nutrition Coordinating Center, Minneapolis, MN).

#### Dietary diversity metrics

We estimated three distinct aspects of dietary diversity: 1) count, 2) evenness of the food intake distribution and 3) the dissimilarity of food items consumed. Count was characterized as the number of food items consumed at least once a week. This is the most commonly used measure of dietary diversity to date. The evenness of the food intake distribution was estimated using the Berry-Index (also known as the Simpson-Index). This index has been widely used as a measure of diversification in economic and ecologic studies, and adopted in recent studies evaluating dietary diversity [16, 21]. The Berry index is defined as $1-\sum_{i=0}^{n}\;s_{i}^{2}$, where s_i_ is the share of food i in the total amount of energy intake and n is the total number of food items. For example, the Berry Index for a participant reporting equal proportions of energy from 20 food items would be 0.95, whereas the Berry Index for a participant with similar energy consumption from 40 food items would be 0.97. To estimate dissimilarity between food items consumed, we used Jaccard distance (JD), a measured adopted in ecological studies to compare species sample sets [22]. The JD between two food items *x* and *y* is defined $\text{as}\;\frac{B_{x}+C_{y}}{A_{xy}+B_{x}+C_{y}}$, where A_xy_ = number of attributes shared by food items x and y; B_x_ = number of attributes unique to x; C_y_ = number of attributes unique to y. Dissimilarity among food items consumed for each individual was estimated by calculating the average distance for pairwise comparisons of all food items consumed by each participant. We selected 12 different food attributes based on likely evidence for effects on of cardio-metabolic health (see Table A in S1 File). Dietary diversity measures were estimated based on total food consumption. We also evaluated whether associations of diversity were heterogeneous by healthfulness by assessing association in healthy and unhealthy food items (see complete description in Table B in S1 File). Theoretical dietary diversity values ranged between 0–120 (food count), and 0–1 (evenness of food distribution and dissimilarity).

#### Measures of dietary quality

We selected quality scores based on scientific evidence for favorable associations with CVD and T2D, including the Dietary Approaches to Stop Hypertension (DASH) score [7, 9, 23], the Alternative Healthy Eating (aHEI) Score [7, 24–26], and an a priori dietary pattern score previously developed in the Coronary Artery Risk Development in Young Adults Study [27]. To estimate DASH scores, participants were categorized into quintiles of consumption of fruits, vegetables, nuts and legumes, low-fat dairy products, whole grains, sodium, sweetened beverages, and red and processed meats[7, 23]. The aHEI Score awards points for higher intakes of fruits, vegetables, nuts and soy, cereal fiber, higher polyunsaturated to saturated fat- and white to red meat ratios, low intakes of trans-fat, dietary supplement use, and moderate alcohol consumption[26]. Finally, the a priori dietary pattern score was created based on intakes of food groups comprising 19 healthy items and 9 unhealthy items. Participants were categorized into quintiles of food intake and assigned 0–4 points for healthy foods, and 4–0 points for increasing quintiles of each unhealthy food intake [27]. A full description of diet quality scores is shown in Table C in S1 File. To ensure variability across 5 levels of consumption, in food groups with substantial number of non-consumers (e.g. avocado, tea), consumers were categorized according to quartiles of intakes, while non-consumers were grouped in one single category. Theoretical dietary quality score values ranged from 8–40 (DASH), 0–87.5 (aHEI), and 0–120 (a priori).

#### Outcomes

We evaluated associations with long-term changes in weight and waist circumference and incident T2D. Weight and waist circumference were measured at each cohort examination by trained personnel using standardized instruments and protocols. Because of a large number of missing values in cohort examination 5 (2010–2011), we assessed changes in anthropometric measurements between cohort examination 4 (2005–2007) and baseline. For this analysis, we excluded subjects with T2D at baseline (n = 859), those with unreliable dietary assessment (incomplete forms, too few or too many foods reported per day, a high frequency of foods skipped, or too many foods coded with the same frequency or serving size) (n = 577), those with daily energy intakes <600 or > 6000 kcal/d (n = 801) and participants with missing information on body weight or waist circumference at cohort examinations 1 or 4 (n = 1,114). In order to minimize confounding from loss of lean muscle mass at older ages or from loss of weight due to chronic disease, we censored data for participants after they reached 65 years of age or if they had been diagnosed with cancer (n = 536), or cardiovascular disease (n = 419). In total, associations of diet diversity and quality with changes in body weight and waist circumference were evaluated in 2,505 MESA participants.

Fasting serum glucose was measured at each examination by rate reflectance spectrophotometry by using thin-film adaptation of the glucose oxidase method (Vitros analyzer; Johnson & Johnson Clinical Diagnostics). New diabetes cases were diagnosed based on new fasting glucose ≥126 mg/dL or the new use of insulin or oral hypoglycemic medications, each assessed at study cohort examinations. After excluding participants with T2D at baseline (n = 859), those with unreliable dietary assessment (n = 577) and those with daily energy intakes <600 or > 6000 kcal/d (n = 801), we included 5,160 participants in this analysis.

#### Covariates

Information on socio-demographic factors, medical history, medication use, lifestyle habits including smoking status and history was obtained at baseline using interviewer-administered and self-completed questionnaires. Physical activity was assessed using the MESA Typical Week Physical Activity Survey[28], a validated semiquantitative questionnaire that captures time and frequency of various physical activities during a typical week in the previous month.

#### Statistical analysis

We evaluated independent associations between measures of diet diversity and quality and 5-year change in waist circumference for each race-ethnicity in MESA using multivariable linear regression with robust variance estimators. We also evaluated associations of diet diversity and quality with incident T2D using Cox proportional-hazards models with time-at-risk until first diagnosis. To understand how intakes of individual food items and selected nutrients were associated with diet diversity and quality, we assessed the partial Spearman correlation adjusting for demographics and lifestyle factors. To assess associations with changes in waist circumference, we used quintiles of the exposures of interest, while associations with T2D were assessed using interquintile median ranges as continuous measures. Because the distribution of evenness was highly skewed, we used natural log-transformed variable for continuous analysis in proportional hazard models with T2D as outcome. The proportional-hazards assumption was not rejected on the basis of Schoenfeld residuals. Also, we found no evidence of nonlinear relationships between each dietary diversity and quality metrics and T2D risk based on restricted cubic spline analysis. To minimize potential confounding, we selected covariates based on their well-established associations with metabolic risk in adults. We imputed missing covariate data (< 2% for most lifestyle factors) using single imputation (SAS proc MI) based on age, sex, race/ethnicity, education, physical activity, BMI, smoking status, LDL-cholesterol, HDL-cholesterol, cholesterol-lowering medication use, and T2D mellitus; results were similar when we used multiple imputation or excluded missing values. Because energy intake is a potential mediator in associations of dietary diversity, we did not include this variable in final statistical models. We evaluated the potential for effect modification by age, race-ethnicity, and BMI. We used two sided p-values with p <0.05 indicating statistical significance. Linear trend was tested by assigning the median value in each quintile to participants and assessing this variable continuously. All analyses were conducted using SAS version 9.3.

## Results

**Fig 1:**
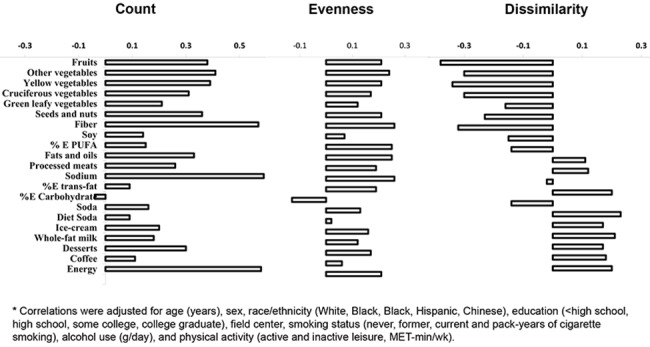
Spearman correlations between dietary factors and diet diversity metrics in 2,505 multi-ethnic US adults.

The mean ±SD age at baseline was 61.9±10.3y, 53% of subjects were female, 40% were overweight and 29% were obese. Most subjects were Whites (43%), followed by Blacks (24.5%), Hispanics (21%) and Chinese Americans (11.5%). The mean ±SD diversity metrics were 32.1±11.0 for food count (range: 3–89 foods), 0.91±0.06 for evenness (range: 0.15–0.98), and 0.71±0.03 for dissimilarity (range: 0.51–0.80). Participants in the higher quintile of evenness of energy distribution were younger, more physically active, more likely to have higher education and were more physically active compared to those in the lowest quintile. Similarly, participants with greater dissimilarity in their diets were younger, more likely to be males, to be Whites, and to have higher education, however those participants also had higher BMI, and were more likely to be smokers compared to those in the lowest quintile of food dissimilarity (Table D in S1 File). In multivariate adjusted analysis, there was a moderate positive correlation between food count and evenness (r = 0.54), while diet dissimilarity showed weak inverse correlations with food count (r = -0.17), and evenness (r = -0.07). Correlation between diet quality and food count or evenness was weak and positive, ranging from 0.03 to 0.20. Conversely, there was an inverse correlation between diet quality scores and diet dissimilarity (Table E in S1 File). When evaluating the top correlations between dietary factors and diversity metrics, there was modest positive correlation between both healthy and unhealthy dietary factors with food count or evenness (Fig 1). On the other hand, consumption of healthy dietary factors (e.g. fruits, vegetables, nuts) showed inverse correlations with food dissimilarity, while consumption of less healthy factors (e.g. soda, trans-fat, desserts) was positively correlated with the same diversity metric.

### Relationships between Dietary Diversity and Quality and 5-year change in Waist Circumference

The mean ±SD 5-y change in waist circumference was 0.78±6.95 cm in Whites, 0.59±7.22 cm in in Blacks, 1.54±6.31 cm in Hispanics, and 1.19±5.14 cm in Chinese Americans. After adjustment for potential confounders, food dissimilarity, but not food count or evenness, was positively associated with gain in waist circumference, with 120% higher gain in participants in the highest quintile of dissimilarity scores. Associations were stronger in Blacks and Chinese, with a 2.5-fold higher gain in waist circumference in in Chinese Americans and a 5-fold higher gain Blacks in the highest quintile of food dissimilarity compared to the lowest category (Table 1). Associations in Blacks were attenuated and no longer statistically significant after further adjustment for diet quality. Associations in other subgroups remained materially unchanged or were strengthened after controlling for diet quality.

**Table 1:** Multivariate-Adjusted 5-year Change in Waist Circumference according to Quintiles of Dietary Diversity Metrics by race-ethnicity in 2,505 US adults.

	Whites (n = 1,057)		Blacks (n = 593)		Hispanics (n = 536)		Chinese (n = 319)		All (n = 2,505)	
	MV Model	MV Model+ aHEI score	MV Model	MV Model+ aHEI score	MV Model	MV Model+ aHEI score	MV Model	MV Model+ aHEI score	MV Model	MV Model+ aHEI score
	Mean (95%) change, lb		Mean (95%) change, lb		Mean (95%) change, lb		Mean (95%) change, lb		Mean (95%) change, lb	
Quintiles of diversity metrics										
Count (food items)										
19	1.7 (0.7,2.7)	1.7 (0.6,2.7)	1.2 (0.0,2.4)	1.3 (0.1,2.5)	2.8 (1.9,3.8)	2.7 (1.7,3.7)	0.9 (-0.1,1.9)	0.5 (-0.1,1.9)	1.7 (1.1,2.3)	1.7 (1.1,2.2)
26	1.3 (0.3,2.2)	1.3 (0.3,2.2)	1.7 (0.3,3.1)	1.7 (0.3,3.1)	2.2 (1.0,3.3)	2.1 (1.0,3.2)	2.5 (1.1,3.8)	0.7 (1.1,3.8)	1.7 (1.1,2.3)	1.7 (1.1,2.3)
31	1.3 (0.5,2.1)	1.3 (0.5,2.1)	1.4 (0.3,2.6)	1.4 (0.3,2.6)	1.3 (0.1,2.4)	1.3 (0.2,2.4)	1.8 (0.5,3.0)	0.6 (0.5,3)	1.4 (0.9,1.9)	1.4 (0.9,1.9)
38	1.7 (0.8,2.5)	1.7 (0.8,2.6)	2.0 (0.5,3.5)	1.9 (0.4,3.4)	1.8 (0.6,3.0)	1.9 (0.7,3.1)	1.4 (0.1,2.7)	0.7 (0.1,2.7)	1.7 (1.1,2.3)	1.7 (1.1,2.3)
47	2.0 (1.1,2.9)	2.0 (1.1,2.9)	0.9 (-0.3,2.1)	0.8 (-0.4,2.0)	2.6 (1.4,3.8)	2.8 (1.6,4.1)	2.2*(1,3.4)	0.6 (1,3.4)	1.9 (1.3,2.4)	1.9 (1.3,2.5)
p-trend	0.56	0.47	0.84	0.70	0.70	0.96	0.34	0.33	0.68	0.66
Evenness (diversification)										
0.85	1.1 (0,2.2)	1.1 (0,2.2)	1.4 (0.1,2.7)	1.4 (0.1,2.7)	2.1 (0.9,3.3)	2 (0.9,3.2)	1.3 (0.2,2.5)	1.3 (0,2.5)	1.4 (0.8,2)	1.4 (0.8,2)
0.91	2.3 (1.4,3.3)	2.3 (1.4,3.3)	0.7 (-0.9,2.3)	0.7 (-0.9,2.3)	2.5 (1.2,3.7)	2.4 (1.2,3.6)	1.5 (0.1,2.9)	1.5 (0,2.9)	1.9 (1.3,2.5)	1.9 (1.3,2.5)
0.93	1.3 (0.5,2.1)	1.3 (0.5,2.1)	2 (0.9,3.1)	2 (0.9,3.1)	2.9 (1.6,4.2)	2.9 (1.6,4.2)	2.2 (1,3.3)	2.2 (0,3.3)	1.9 (1.4,2.4)	1.9 (1.4,2.4)
0.94	1.6 (0.8,2.4)	1.6 (0.8,2.4)	2.4 (0.9,4)	2.4 (0.9,4)	1.9 (1,2.8)	2 (1,2.9)	1.6 (0.4,2.9)	1.6 (0,2.9)	1.8 (1.3,2.4)	1.8 (1.3,2.4)
0.96	1.4 (0.5,2.3)	1.4 (0.5,2.3)	0.8 (-0.2,1.9)	0.8 (-0.2,1.8)	1.5 (0.5,2.5)	1.5 (0.5,2.6)	1.7 (0.6,2.9)	1.7 (0,2.9)	1.3 (0.8,1.8)	1.3 (0.8,1.8)
p-trend	0.64	0.60	0.82	0.89	0.56	0.67	0.60	0.60	0.64	0.62
Dissimilarity										
0.67	2.3 (1,3.6)	1.6 (0.3,3)	0.4 (-1.1,1.8)	0.3 (-1.3,1.8)	1.8 (0.7,2.9)	0.9 (-0.3,2.0)	0.8 (-0.2,1.9)	0.6 (-0.3,1.6)	0.9 (0.3,1.6)	0.9 (0.2,1.5)
0.70	1 (0,2.1)	1.5 (0.6,2.5)	1.1 (-0.4,2.6)	1.2 (-0.1,2.4)	1.8 (0.7,3)	1.5 (0.3,2.7)	1.3 (0.3,2.2)	1.9 (1,2.8)	1.5 (1,2.1)	1.5 (1,2.1)
0.71	1.8 (0.9,2.7)	1 (0.1,1.9)	1.3 (-0.1,2.8)	1.3 (-0.2,2.7)	1.8 (0.7,3)	2.7 (1.7,3.8)	1.6 (0.4,2.8)	2.7 (1.4,4)	1.7 (1.1,2.2)	1.7 (1.1,2.2)
0.73	1.5 (0.7,2.3)	1.6 (0.8,2.4)	1.5 (0.3,2.7)	2.2 (1,3.3)	2.7 (1.5,3.9)	2.8 (1.7,3.9)	3.4 (2.1,4.8)	1.9 (0.4,3.5)	2.0 (1.5,2.5)	2.0 (1.5,2.5)
0.75	1.7 (0.9,2.6)	1.9 (1.2,2.7)	2.5 (1.2,3.7)	1.6*(0.3,3)	3.1 (1.8,4.3)	2.6 (1.4,3.8)	2.8* (1.1,4.5)	3.8*(1.6,6.1)	2.0 (1.5,2.6)	2.1 (1.5,2.6)
p-trend	0.62	0.58	0.047	0.15	0.12	0.02	0.01	0.004	0.008	0.01

Values are multivariate-adjusted mean (95%CI). MV model included age (years), sex, race/ethnicity (White, Black, Black, Hispanic, Chinese), education (<high school, high school, some college, college graduate), field center, smoking status (never, former, current and pack-years of cigarette smoking), alcohol use (g/day), and physical activity (active and inactive leisure, MET-min/wk).

*Statistically significant difference from the lower quintile (p-value < 0.05)

In multivariate adjusted analysis, higher DASH scores were associated with lower gain in waist circumference in Blacks and Hispanics, but not in Whites or Chinese Americans. There was no association between the AHEI or a priori diet quality scores and change in waist circumference after 5y (Table 2).

**Table 2:** Multivariate-Adjusted 5-year Change in Waist Circumference according to Quintiles of Diet Quality Scores by race-ethnicity in 2,505 US adults.

	Whites (N = 1,057)	Blacks (N = 593)	Hispanics (N = 536)	Chinese (N = 319)	All (N = 2,505)
	Mean change (95%CI), cm	Mean change (95%CI), cm	Mean change (95%CI), cm	Mean change (95%CI), cm	Mean change (95%CI), cm
Quintiles of diet quality scores					
DASH					
18	1.5 (0.7,2.3)	1.9 (0.5,3.8)	2.7 (1.8,3.6)	2.0 (0.9,3.0)	1.9 (1.4,2.4)
22	1.0 (0.1,2.0)	2.2 (0.8,3.6)	3 (1.8,4.1)	1.6 (0.6,2.6)	1.9 (1.3,2.4)
25	1.9 (1.1,2.8)	0.9 (-0.5,2.2)	2.1 (1.1,3.1)	1.5 (0.5,2.5)	1.7 (1.2,2.2)
28	1.7 (0.9,2.6)	1.4 (-0.2,3.0)	0.5 (-0.9,1.8)	1.3 (-0.1,2.7)	1.4 (0.7,2.0)
32	1.4 (0.5,2.3)	-0.1* (-1.7,1.5)	2.1 (0.6,3.6)	2.3 (0,4.5)	1.3 (0.6,2.0)
p-trend	0.81	0.06	0.06	0.81	0.09
AHEI					
21	1.7 (0.9,2.6)	0.2 (-1.1,1.4)	2.9 (2.0,3.9)	1.9 (0.6,3.3)	1.7 (1.1,2.2)
28	1.7 (0.8,2.6)	2.1 (0.7,3.5)	1.8 (0.8,2.8)	1.5 (0.4,2.6)	1.7 (1.2,2.3)
31	1.4 (0.6,2.2)	2.1 (0.8,3.3)	2.3 (1.4,3.2)	1.3 (0.2,2.5)	1.8 (1.2,2.3)
36	1.4 (0.4,2.3)	1.9 (0.7,3.1)	0.8 (-0.5,2.2)	2.3 (1.2,3.3)	1.5 (0.9,2.1)
44	1.5 (0.5,2.4)	1.0 (-0.2,2.3)	2.1 (0.4,3.9)	1.3 (0.1,2.6)	1.6 (1.0,2.2)
p-trend	0.61	0.52	0.12	0.79	0.67
A priori					
37	1.3 (0.3,2.2)	1.3 (0.3,2.2)	2.6 (1.6,3.6)	2.8 (0.9,4.8)	1.6 (1.0,2.1)
46	1.6 (0.5,2.6)	2.0 (0.7,3.3)	2.3 (1.5,3.2)	1.3 (0.1,2.6)	1.9 (1.4,2.5)
53	1.9 (1.0,2.8)	1.6 (0.2,3.0)	1.6 (0.5,2.7)	1.7 (0.8,2.7)	1.8 (1.2,2.3)
60	1.6 (0.8,2.5)	1.5 (-0.1,3.0)	1.7 (0.4,3.1)	1.9 (1.0,2.8)	1.7 (1.1,2.2)
70	1.4 (0.5,2.2)	0.2 (-1.5,1.9)	2.5 (0.9,4.1)	1.0(-0.4,2.4)	1.3 (0.7,2.0)
p-trend	0.94	0.60	0.56	0.42	0.50

Values are multi-variate adjusted mean (95%CI). MV model included age (years), sex, race/ethnicity (Whites, Black, Black, Hispanic, Chinese), education (<high school, high school, some college, college graduate), field center, smoking status (never, former, current and pack-years of cigarette smoking), alcohol use (g/day), and physical activity (active and inactive leisure, MET-min/wk).

*Statistically significant difference when compared to the lower quintile (p-value < 0.05

### Relationships between Dietary Diversity and Quality and T2D

During approximately 10-y of follow-up, 588 new cases of T2D were diagnosed. Incidence rates per 10,000 person-years were 84 in Whites, 140 in Blacks, 162 in Hispanics, and 113 in Chinese participants. After adjusting for potential confounders, we found no association between food count, evenness or dissimilarity and risk of T2D in multi-ethnic US adults (Table 3). Further adjustment for diet quality (a priori scores) did not materially change measures of association with T2D. Associations were similar in men and women (data not shown).

**Table 3:** HRs (95% CIs) of type II diabetes for 1-interquintile range (IQR) unit of dietary diversity metrics in 5,160 U.S. adults.

	Whites	Blacks	Hispanics	Chinese	All
case/person-years	192/22,814	166/11,882	162/10,024	68/6,004	588/55,724
Food items (IQR = 28)					
Multivariate model	0.88 (0.53,1.45)	1.29 (0.82,2.04)	0.81 (0.5,1.31)	1.05 (0.47,2.31)	0.98 (0.76,1.27)
Multivariate model + a priori score	1.04 (0.61,1.78)	1.33 (0.83,2.11)	0.82 (0.5,1.35)	1.11 (0.51,2.43)	1.06 (0.82,1.38)
Evenness (IQR = 0.10)					
Multivariate model	1.09 (0.82,1.45)	1.14 (0.85,1.54)	0.99 (0.7,1.41)	0.79 (0.52,1.2)	1.06 (0.9,1.24)
Multivariate model + a priori score	1.12 (0.84,1.49)	1.14 (0.85,1.54)	1 (0.7,1.41)	0.81 (0.53,1.24)	1.07 (0.91,1.25)
Dissimilarity (IQR = 0.08)					
Multivariate model	1.14 (0.74,1.76)	1.3 (0.85,1.97)	1.04 (0.66,1.63)	1.54 (0.86,2.75)	1.16 (0.92,1.45)
Multivariate model + a priori score	1.01 (0.64,1.6)	1.32 (0.84,2.07)	1 (0.62,1.63)	1.45 (0.8,2.64)	1.08 (0.85,1.37)

Values are HR (95%CI). Multivariate-adjusted models include age (years), sex, race/ethnicity (Whites, Black, Black, Hispanic, Chinese), education (<high school, high school, some college, college graduate), energy intake (kcal/day), field center, smoking status (never, former and current smoker, and pack/years of cigarette smoking), alcohol use (g/day), physical activity (active and inactive leisure, MET-min/wk), dietary supplement use (yes/no)

On the other hand, we found an inverse association between the AHEI and a priori scores and T2D, with each interquintile median range unit increase associated with 24% (AHEI) and 27% (a priori) lower risk of T2D respectively (Table 4). Associations of diet quality were stronger in Whites and Chinese Americans, although confidence intervals for the latter group were broad. Associations of diet quality were attenuated after adjustment for potential mediators such as baseline BMI and waist circumference. All findings were generally similar in men versus women (not shown).

**Table 4:** HRs (95% CIs) of type II diabetes per 1-interquintile rage (IQR) unit of diet quality scores in 5,160 U.S. adults.

	Whites	Blacks	Hispanics	Chinese	All
case/person-years	192/22,814	166/11,882	162/10,024	68/6,004	588/55,724
DASH score (IQR = 14)					
Multivariate model^1^	0.74 (0.48,1.14)	0.79 (0.52,1.22)	1.21 (0.73,1.99)	0.69 (0.27,1.75)	0.85 (0.66,1.09)
Multivariate model^2^	0.92 (0.60,1.42)	0.93 (0.60,1.44)	1.36 (0.82,2.20)	0.64 (0.24,1.67)	1.02 (0.79,1.30)
AHEI Score (IQR = 23)					
Multivariate model^1^	0.63 (0.43,0.92)	0.84 (0.57,1.24)	0.89 (0.57,1.38)	0.75 (0.38,1.47)	0.76 (0.61,0.94)
Multivariate model^2^	0.70 (0.47,1.03)	0.89 (0.60,1.32)	0.93 (0.60,1.45)	0.73 (0.37,1.45)	0.81 (0.65,1.00)
A priori score (IQR = 33)					
Multivariate model^1^	0.64 (0.42,0.96)	0.83 (0.54,1.29)	0.91 (0.56,1.49)	0.57 (0.21,1.59)	0.73 (0.57,0.94)
Multivariate model^2^	0.81 (0.53,1.23)	1.02 (0.66,1.59)	1.03 (0.63,1.69)	0.57 (0.20,1.60)	0.91 (0.71,1.17)

Values are HR (95%CI). Multivariate-adjusted model 1 included age (years), sex, race/ethnicity (White, Black, Black, Hispanic, Chinese), education (<high school, high school, some college, college graduate), field center, smoking status (never, former and current smoker, and pack/years of cigarette smoking), energy (kcal/day), alcohol use (g/day), physical activity (active and inactive leisure, MET-min/wk), dietary supplement use (yes/no). Multivariate model 2 included further adjustment for BMI (kg/m2) and baseline waist circumference (cm).

### Associations of diet diversity metrics for healthy vs. unhealthy foods

When we stratified foods based on healthfulness, we found overall no association of diet diversity metrics and change in waist circumference (Table G in S1 File), however there were few exceptions. Hispanic and Chinese participants reporting higher dissimilarity in healthy food consumption showed greater gain in waist circumference after 5y. There was no association of diversity metrics of healthy and unhealthy foods with risk of T2D in this multi-ethnic cohort. We also evaluated associations of food count restricted only to fruits and vegetables (N = 23 items), and found no associations with either change in waist circumference or incident T2D (not shown).

## Discussion

In this large prospective cohort of multiethnic Americans, we found no association between two established measures of dietary diversity, food count or evenness, and change in WC or risk of T2D. Higher dissimilarity in food consumption, a third metric of diet diversity, was actually associated with higher gain in WC after 5y. In contrast, diet quality scores, as defined by the aHEI the a priori scores, showed inverse associations with risk of T2D. When we restricted diet diversity metrics to only more healthful or unhealthy foods, no significant associations were seen with either WC or incident T2D. To our knowledge, this is the first time that multiple metrics of diet diversity were systematically characterized and their relations with metabolic health prospectively evaluated in a multi-ethnic population. Our findings do not support the prevailing notion of diet diversity leading to a healthier diet or lower metabolic risk. Indeed, our results show inverse correlations between dietary dissimilarity and diet quality, and positive associations with metabolic risk.

Greater food count and evenness were associated with higher intakes of both healthy and unhealthy foods, resulting in weak correlations with overall diet quality scores. This suggests that greater diversity, as measured by either count or evenness, leads to increased intakes of both healthier and unhealthy foods. Thus, potential benefits of increased intakes of fruits and vegetables may be outweighed by unfavorable effects of trans-fat, sodium, starch and refined carbohydrates, resulting in no overall benefit to metabolic health. Although greater food count could help increase nutrient adequacy in low-income populations, particularly those consuming most calories from a limited number of staple foods [29] our findings show no benefit to diet quality or diet healthfulness associated with increased food count or with a more even distribution of energy across foods. Interestingly, greater dissimilarity among foods—an aspect of diet diversity that captures diversity of food attributes—was associated with lower intakes of healthy foods and higher intakes of unhealthy foods. This would explain the positive association of diet dissimilarity with gain in abdominal obesity. Notably, even when restricting to only generally healthful foods, no independent association with gain in WC nor incident T2D was seen with any of the metrics of dietary diversity.

One of the novel contributions of our analysis is the characterization of multiple different metrics of diversity, each capturing distinct aspects of variety in the diet. Food count, which has been widely used in prior studies, only evaluates the number of different types of foods. For example, using the analogy of wildlife habitats, count would provide a crude measure of the number of different species present in an ecosystem. We also evaluated evenness, which captures the distribution of intake across different foods consumed. For example, an ecosystem could contain many different species but be dominated by one or two (low evenness), or contain the same number of species that are more evenly distributed (high evenness). Third, we evaluated dissimilarity, which captures diversity in types of foods consumed based on shared or unique attributes relevant to metabolic health (S1 Fig). For instance, an ecosystem could contain multiple species that are relatively similar to each other, such as seen in temperate forests, or in contrast multiple species that are widely varying, such as seen in tropical rainforests. Most prior studies have only used food count to understand the role of diet diversity, and evaluating restricted subgroups of the diet (e.g., only fruits and vegetables). Our assessment of three different diversity metrics and the entire diet provide the most comprehensive assessment to-date of how diet diversity relates to healthful dietary patterns and metabolic outcomes.

Few previous studies have evaluated diet diversity and central obesity, generally evaluating only food counts of specific food groups, assessing cross-sectional relationships, and including relatively small populations. [13, 30–32] Consistent with our results, three of these studies found positive associations between diet diversity and body fat metrics in adult populations[13, 30, 31]. Also in agreement with our findings, Bezerra and Sichieri reported that greater diet diversity, based on food count, was associated with higher consumption of unhealthy foods such as sweets, sugar-sweetened beverages, crackers, cookies and cakes in Brazil [31]. Azadbakht and Esmaillzadeh reported inverse cross-sectional associations between dietary counts and prevalence of higher waist circumference and obesity in young female Iranians[32]; however this study was small (N = 289), based on a student population, evaluated only five food groups (bread-grains, vegetables, fruits, meats, dairy), and could not evaluate temporality of associations. Altogether, observations from our study and other prior investigations suggest that overall diet diversity is linked to less healthy dietary patterns and higher risk of obesity.

To our knowledge, only one prior study has evaluated associations of diet diversity and risk of T2D. Based on a case-cohort study of 3,704 Europeans, Cooper et. al found 39% lower risk of T2D with higher counts of fruits and vegetables[12], independent of the total quantity of fruits and vegetables consumed. This prior study did not evaluate diversity of other foods consumed nor other metrics of diversity. In sensitivity analysis, we found no significant association of greater diversity of fruits and vegetables with risk of T2D. Our findings build upon and considerably expand these prior studies by investigating multiple metrics of diet diversity, including a large number of multiethnic participants, and prospectively evaluating associations with both central obesity and T2D.

Consistent with previous studies, we found inverse associations or trends toward inverse associations between dietary quality and risk of T2D [7, 33, 34]. Our findings, the first from a multi-ethnic US population, suggest benefits of diet quality for T2D in most race-ethnicities. The reasons for the absence of significant association in Hispanics requires further investigation; this could be due to dietary and/or other lifestyle factors that may be unique to Hispanics, or be a chance finding. Across different diet pattern scores, associations were generally strongest for the AHEI score, suggesting potential additional benefits from higher consumption of polyunsaturated fatty acids, white meat, and moderate alcohol consumption.

Our study has several strengths. The use of novel metrics of diet diversity using all food items allowed us a comprehensive evaluation of diet diversity in our cohort. Information on waist circumference and T2D incidence was prospectively collected, minimizing concerns with reverse causality. Compared to more homogeneous cohorts, the multi-ethnic nature of MESA allowed the study of a wider range of dietary behaviors and susceptibility to metabolic outcomes, increasing confidence in the validity and generalizability of the results.

Our study also has potential limitations. As previously mentioned, the use of FFQ to assess food diversity may have limited our ability to fully capture diet diversity, especially in subgroup analysis. In addition, potential random measurement error in dietary assessment could have attenuated measures of association toward the null. Hence, our results may have underestimated true relations. On the other hand, the FFQ-derived measures of diet quality did predict risk of diabetes, which may suggest that measurement error alone may not fully explain the absence of association with food count or evenness. Finally, although we carefully adjusted for major potential confounders, we cannot exclude the possibility of residual confounding. However, our observed associations were robust to adjustment for several potential risk factors.

In conclusion, our findings provide little evidence for benefits of dietary diversity for either waist circumference or T2D. Greater dissimilarity among foods was actually positively associated with increase in waist circumference. Our results challenge the notion that “eating everything in moderation” leads to greater diet quality or better metabolic health. Our findings support the importance of diet quality, independent of diversity, and highlight the need for greater investigation of relationships between diet diversity and metabolic health in understudied populations.

## Supporting Information

S1 Fig Jaccard index of dissimilarity between broccoli and selected food items. Jaccard distance (JD) between two food items x and y is defined as (B_x+C_y)/(A_xy+B_x+C_y), where Axy = number of attributes shared by food items x and y; Bx = number of attributes unique to x; Cy = number of attributes unique to y. Food attributes: plant or animal food, PUFA-, antioxidant-, fiber-, alcohol-, and sodium content (high or moderate), glycemic load, food processing, fermentation and food structure (solid or liquid)
(TIF)

S1 File Attributes of food dissimilarity scores (Table A). Food items in the MESA FFQ stratified by healthfulness (Table B). Description of diet quality score components (Table C). Baseline characteristics of 5,160 US adults free of diabetes at baseline by dietary diversity measures in the Multi-Ethnic Study of Atherosclerosis (Table D). Partial Spearman correlations between dietary diversity measures and diet quality scores in 5,160 participants (Table E). Baseline characteristics of 5,160 US adults free of diabetes at baseline by dietary quality scores in the Multi-Ethnic Study of Atherosclerosis (Table F). Multivariate-Adjusted 5-year Change in Waist Circumference according to Quintiles of Dietary Diversity of healthy and unhealthy food items by race-ethnicity in 2,505 US adults (Table G). HRs (95% CIs) of type II diabetes for 1-interquintile rage (IQR) unit of dietary diversity of healthy and unhealthy foods in 5,160 U.S. adults (Table H). (DOCX)
