# Supplementary figures and images for: Everything in Moderation - Dietary Diversity and Quality, Central Obesity and Risk of Diabetes
Source: PLoS One. 2015 Oct 30;10(10):e0141341. doi: 10.1371/journal.pone.0141341 (PMC4627729; doi:10.1371/journal.pone.0141341)

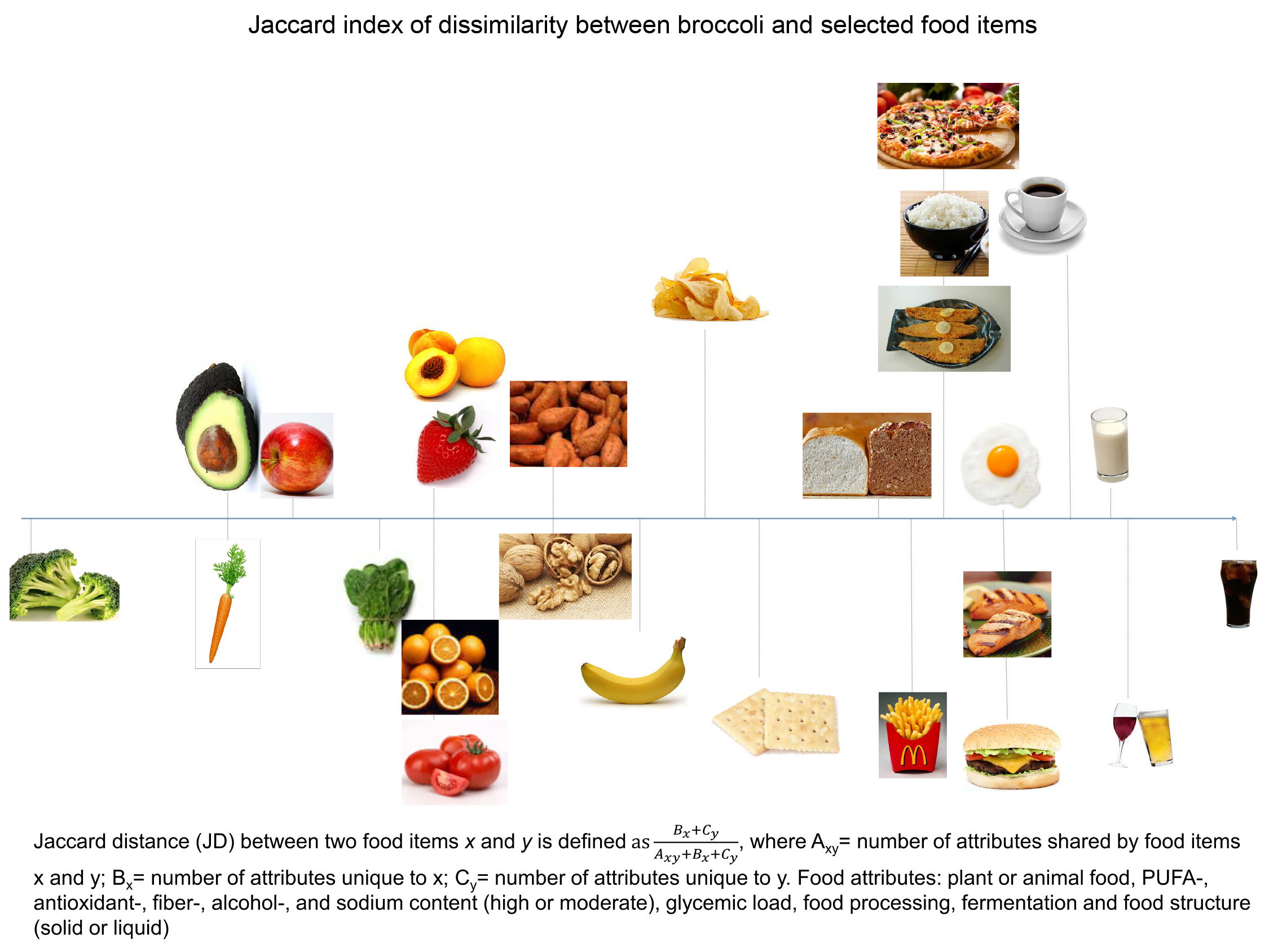

Supplement: S1 Fig — Jaccard distance (JD) between two food items x and y is defined as (B_x+C_y)/(A_xy+B_x+C_y), where Axy = number of attributes shared by food items x and y; Bx = number of attributes unique to x; Cy = number of attributes unique to y. Food attributes: plant or animal food, PUFA-, antioxidant-, fiber-, alcohol-, and sodium content (high or moderate), glycemic load, food processing, fermentation and food structure (solid or liquid) (TIF) [file pone.0141341.s001.tif]
