# Supplementary material for: Everything in Moderation - Dietary Diversity and Quality, Central Obesity and Risk of Diabetes
Source: PLoS One. 2015 Oct 30;10(10):e0141341. doi: 10.1371/journal.pone.0141341 (PMC4627729; doi:10.1371/journal.pone.0141341)
Supplement: S1 File — (DOCX) [file pone.0141341.s002.docx]

Table A - Attributes of food dissimilarity scores

| Attribute | Categories | Criterion |
| --- | --- | --- |
| Food type | Animal  Plant | Main protein source |
| Food processing | Highly processed  Moderately processed  Minimally processed |  |
| Food structure | Liquid  Solid |  |
| Fermentation | Yes  No |  |
| Caloric liquid | Yes  No |  |
| Alcohol content | Yes  No |  |
| Fiber content | High  Moderate  Low | tertiles of total fiber content per 100g of food |
| Total PUFA content | High  Moderate  Low | tertiles of PUFA content per 100g of food |
| EPA+DHA content | High | top tertile of EPA+DHA per 100g of food |
| Glycemic load (GL) | High | top tertile of GL |
| Antioxidants content | High  Moderate  Low | tertiles of antioxidant scores of food items based on nutrient content (vitamins A, C and E; β-, γ- and δ-tocopherol; β and α- carotene, lycopene and lutein([33](#_ENREF_33))) |
| Total trans-fat (TFA) content | High | >0.5g TFA per 100g of food |
| Sodium content | High  Moderate  Low | tertiles of sodium content per 100g of food |
| Glycemic load (GL) | High  Low | top tertile of GL (high) |
| Antioxidants content | High  Moderate  Low | tertiles of food antioxidant scores based on nutrient content (vitamins A, C and E; β-, γ- and δ-tocopherol; β and α- carotene, lycopene and lutein([33](#_ENREF_33))) |

Table B - Food items in the MESA FFQ stratified by healthfulness

|  | Summary description | FFQ items |
| --- | --- | --- |
| Healthy foods (49 items) | Fruits, all vegetables (green-leafy, cruciferous, dark-yellow, tomatoes), vegetable soups, legumes, seafood, whole grains, nuts and seeds, high-fiber breakfast cereals, brown or wild rice, margarine or mayonnaise on bread or vegetables, yogurt, regular- and low-fat cheese, coffee, herbal, black or green tea, alcoholic drinks | - peaches, apricots, nectarines, plums - cantaloupe, mango, papaya - strawberries, blueberries, other berries - apple, applesauce, pears, - bananas, plantains, - oranges, grapefruit, tangerines, kiwi, - dried fruits (raisins, prunes, figs, apricots) - other fruits (pineapple, persimmon, grapes etc) - tossed salad with lettuce - tossed salad with spinach or dark greens - tomatoes (cooked or raw) and tomato juice - avocado, guacamole - carrots - broccoli, cabbage, cauliflower, Brussel sprouts, sauerkraut, kimchee - green beans, peas, snow peas - corn, hominy - winter squash, acorn squash - pinto, black, baked, butter or red beans - any other vegetable - pea, lentil, black bean, potajes soup - miso soup or sauce with soybean paste - salsa, pico de gallo - brown or wild rice - sweet potatoes, yams - dark, whole grain breads or rolls - oatmeal - high fiber cold cereal - almonds, walnuts, pecans, other nuts - sunflower, pinyon, other seeds - peanuts, peanut butter - Fried fish or fish sandwich, fried shrimp, calamari - Shrimp, lobster, crab, oysters, mussels (not fried) - Tuna, salmon, sardines - Other broiled, steamed, baked or raw fish - Stir-fried shrimp or fish with vegetables, - Stir-fried tofu or tempeh with vegetables - Stir-fried vegetables (no meat) - Fish stew or seafood gumbo, paella, - Cottage or ricotta cheese - Regular cheese - Plain yogurt - Flavored yogurt - margarine or mayonnaise on bread or vegetables - coffee (regular or decaf), - herbal tea - black or green tea - wine - beer - liquor or mixed drinks |
| Unhealthy foods (33 items) | processed and unprocessed red meat, white bread or rolls, low-fiber cereals, salty snacks, white rice, potatoes, French fries, bakery desserts, candy, sugar sweetened beverages | - sausage, chorizo, scrapple, bacon - beef, pork or lamb steaks, roasts, barbecue or ribs - hamburger, cheeseburger, meat loaf - ham hocks, pig's feet, chicarones - ham, hot dogs, bologna, salami, other lunch meats - liver including chicken liver, other organ meats - gravies made with meat or poultry drippings - white, Mexican or sticky rice - Fried rice - French fries, fried potatoes, hash browns - Boiled, baked or mashed potatoes - potato or corn or tortilla chips - crackers, pretzels, popcorn - flour or corn tortilla on the side - Pizza - pancakes, waffles, French toast - other hot cereal (grits, cream of wheat etc) - low-fiber cold cereal - White bread or rolls - bran muffins - biscuits, croissants, corn bread, hush puppies - White doughnuts, cookies, cakes, pastries, pop tarts, Chinese or Mexican desserts - chocolate doughnuts, cookies, cakes, brownies or candy - Sugar, jelly, jam or molasses on bread or cereal - regular ice cream - dessert made with tofu - frozen yogurt, low-fat ice cream, ice milk, sorbet - Pies - pudding, custard, flan - sweetened condensed milk - other candy including hard candy and licorice - regular soft drinks, soda, sweetened mineral water - hot chocolate milk |
| Mixed dishes or unknown effects (40 items) | mixed dishes, fruit juices, whole and low-fat milk, butter, eggs, chicken, breakfast mixes, diet soft drinks, non-alcoholic beer, | - oriental noodles with meat - Chinese dumplings, spring roll, dim sum, - stir-fried beef, pork or chicken with vegetables - Chow mein - cream soups (chowders, potato and cheese soup) - burritos, quesadillas or fajitas with meat - burritos, quesadillas or fajitas without meat - enchiladas, tamales, tacos or nachos with meat - enchiladas, tamales, tacos or nachos without meat - picadillo, carne guisada, menudo - arroz con pollo - chili with meat and beans - red chili with meat - green chili with meat - refried beans - pasta with cream or cheese - pasta with cream sauce and meat - pasta with tomato sauce - pasta with tomato sauce and meat - meat, chicken or turkey stew, pot pie or empanada - chicken salad - pasta salad; - roasted broiled or baked chicken or turkey - fried chicken - Other soup including egg drop and chicken noodle - Eggs, homelets, huevos rancheros - Orange juice, grapefruit juice - Any other fruit juice - unsweetened mineral water, - whole milk - 2% milk - Skim, 1% or butter milk - Soy milk - instant breakfast, Ensure, Slimfast - butter on bread or vegetables - diet soft drinks - non-alcoholic beer - Milk in coffee or tea - Cream in coffee or tea - Sugar or honey in coffee or tea |

Table C – Description of diet quality score components

| Diet quality score | Components | Maximum score |
| --- | --- | --- |
| DASH ^17^ | Fruits | Top quintile |
|  | Vegetables | Top quintile |
|  | Nuts and legumes | Top quintile |
|  | Whole grains | Top quintile |
|  | Low-fat dairy | Top quintile |
|  | Sodium | Bottom quintile |
|  | Red and processed meats | Bottom quintile |
|  | Sweetened beverages | Bottom quintile |
| Alternative Healthy Eating Score ^15^ | Vegetables | 5 serving/d |
|  | Fruits | 4 serving/d |
|  | Nuts and soy protein | 1 serving/d |
|  | White:red meat | 4 |
|  | Cereal fiber | 15 g/d |
|  | trans-fat | ≤ 0.5% energy |
|  | PUFA:SFA | ≥ 1 |
|  | Duration of vitamin use | ≥ 5y |
|  | Alcohol | Men: 1.5 – 2.5 serving/d  Women: 0.5 – 1.5 serving/d |
| A priori dietary pattern score([26](#_ENREF_26)) | 42 food groups  19 healthy foods (avocado, legumes, beer, coffee, fatty fish (not fried), Fruit, Green-leafy vegetables, yellow vegetables, tomato, other vegetables, Lean fish (not fried), Low-fat dairy, liquor, poultry, seeds and nuts, soy products, tea, whole grain, wine);  11 unhealthy foods (butter  fried poultry, fried fish, fried potato, red meat, processed meat, bakery desserts, salty snacks, soft drinks, candy, whole fat dairy); 12 neutral foods (chocolate, diet soda, eggs, fruit juice, margarine, meal replacements, pickled food, potatoes, refined grains, shellfish, soups, sugar substitutes) | 0-4 for for healthy food items; 4-0 for unhealthy food items |

Table D - Baseline characteristics of 5,160 US adults free of diabetes at baseline by dietary diversity measures in the Multi-Ethnic Study of Atherosclerosis

|  | **Foods per week** | | | **Evenness** | | | **Dissimilarity** | | |
| --- | --- | --- | --- | --- | --- | --- | --- | --- | --- |
|  | Q1 | Q3 | Q5 | Q1 | Q3 | Q5 | Q1 | Q3 | Q5 |
| Median | 19 | 31 | 47 | 0.85 | 0.93 | 0.96 | 0.67 | 0.71 | 0.75 |
| **Age**, years | 61.1 (10.0) | 62.8 (10.4) | 61.4 (10.9) | 62.1 (10.2) | 62.3 (10.4) | 61 (10.3) | 63.8 (10.4) | 62.6 (10.5) | 60.1 (9.8) |
| **Sex**, % male | 47.4 | 46.4 | 46.7 | 49.2 | 46.4 | 44.0 | 36.7 | 46.2 | 54.9 |
| **Whites,** % | 36.6 | 46.0 | 40.4 | 42.7 | 45.7 | 40.3 | 30.8 | 45.0 | 50.2 |
| **Blacks,** % | 27.4 | 23.0 | 27.6 | 24.5 | 22.6 | 29.0 | 21.6 | 24.5 | 26.3 |
| **Hispanics,** % | 21.3 | 20.7 | 23.4 | 18.9 | 19.6 | 21.0 | 21.1 | 21.6 | 19.9 |
| **Chinese,** % | 14.7 | 10.4 | 8.5 | 14.0 | 12.1 | 9.7 | 26.4 | 8.9 | 3.6 |
| **Education**, some college degree % | 62.9 | 64.7 | 68.1 | 62.8 | 65.3 | 71.6 | 60.4 | 64.4 | 72.1 |
| **Current smoking**, % | 14.0 | 11.9 | 13.4 | 10.9 | 12.6 | 13.4 | 5.6 | 11.9 | 18.4 |
| **Alcohol intake**, g/day | 4.4 (11.3) | 5.8 (11.7) | 6.2 (13) | 5.7 (15.5) | 5.8 (12.6) | 5.6 (9.3) | 2.1 (7.6) | 5.4 (12.8) | 10.2 (17.5) |
| **Body mass index**, kg/m2 | 28 (5.6) | 28 (5.1) | 28.1 (5) | 28 (5.5) | 27.9 (5.2) | 28.2 (5.3) | 26.5 (4.6) | 28.2 (5.3) | 28.6 (5.4) |
| **% Lean** | 31.6 | 28.3 | 28.1 | 31.4 | 31.4 | 30.1 | 39.9 | 28.5 | 26.4 |
| **% Overweight** | 39.0 | 41.7 | 41.4 | 39.1 | 40.6 | 39.2 | 39.4 | 41.6 | 41.7 |
| **% Obese** | 29.4 | 30.1 | 30.6 | 29.5 | 27.9 | 30.6 | 20.8 | 29.9 | 31.9 |
| **Physical Activity**, MET-min/week | 2536 (3581) | 2434 (2873) | 2666 (3111) | 2363 (2995) | 2352 (2628) | 2850 (3343) | 2443 (3057) | 2467 (2928) | 2590 (3255) |
| **Dietary factors** |  |  |  |  |  |  |  |  |  |
| **Fruit intake,** serving/day | 1.3 (1.3) | 2.1 (1.6) | 2.7 (1.7) | 1.6 (1.4) | 2.1 (1.6) | 2.5 (1.8) | 2.8 (2.0) | 2.1 (1.5) | 1.1 (1.0) |
| **Vegetables,** serving/day | 1.6 (1.3) | 2.5 (1.3) | 3.5 (1.7) | 2 (1.3) | 2.5 (1.5) | 3.2 (1.8) | 3.2 (1.8) | 2.6 (1.5) | 1.7 (1.1) |
| **Whole grain,** serving/day | 0.6 (0.6) | 0.8 (0.7) | 1.0 (0.7) | 0.8 (0.8) | 0.8 (0.7) | 0.8 (0.7) | 0.9 (0.8) | 0.8 (0.7) | 0.7 (0.7) |
| **Fish,** serving/day | 0.2 (0.2) | 0.3 (0.3) | 0.5 (0.4) | 0.2 (0.2) | 0.3 (0.3) | 0.4 (0.4) | 0.3 (0.3) | 0.3 (0.3) | 0.3 (0.3) |
| **Unprocessed Meat,** serving/day | 0.3 (0.3) | 0.4 (0.3) | 0.7 (0.5) | 0.3 (0.3) | 0.4 (0.3) | 0.6 (0.5) | 0.4 (0.4) | 0.5 (0.4) | 0.4 (0.3) |
| **Processed Meat,** serving/day | 0.2 (0.3) | 0.2 (0.3) | 0.3 (0.3) | 0.2 (0.3) | 0.2 (0.3) | 0.3 (0.3) | 0.1 (0.2) | 0.2 (0.3) | 0.2 (0.3) |
| **Refined grains,** serving/day | 1.0 (0.8) | 1.2 (0.9) | 1.7 (1.1) | 1.1 (0.9) | 1.3 (1) | 1.4 (0.9) | 1.2 (1) | 1.3 (1) | 1.1 (0.8) |
| **Soda,** serving/day | 0.4 (1) | 0.4 (1) | 0.5 (1) | 0.5 (1.3) | 0.4 (0.9) | 0.4 (0.6) | 0.1 (0.6) | 0.5 (1.1) | 0.5 (1) |

Q2 and Q4 are not shown for space limitations

Table E – Partial Spearman correlations between dietary diversity measures and diet quality scores in 5,160 participants

|  | Evenness | Dissimilarity | DASH | AHEI | A priori |
| --- | --- | --- | --- | --- | --- |
| Count | 0.54 | -0.17 | 0.17 | 0.20 | 0.18 |
| Evenness |  | -0.07 | 0.06 | 0.04 | 0.03 |
| Dissimilarity |  |  | -0.37 | -0.34 | -0.37 |
| DASH |  |  |  | 0.54 | 0.78 |
| AHEI |  |  |  |  | 0.56 |

Correlations were adjusted for age (years), sex, race/ethnicity (White, Black, Black, Hispanic, Chinese), education (<high school, high school, some college, college graduate), field center, smoking status (never, former, current and pack-years of cigarette smoking), alcohol use (g/day), and physical activity (active and inactive leisure, MET-min/wk).

Table F - Baseline characteristics of 5,160 US adults free of diabetes at baseline by dietary quality scores in the Multi-Ethnic Study of Atherosclerosis

|  | **DASH** | | | | **AHEI** | | | | **A Priori** | | | |
| --- | --- | --- | --- | --- | --- | --- | --- | --- | --- | --- | --- | --- |
|  | Q1 | | Q5 | | Q1 | | Q5 | | Q1 | | Q5 | |
|  | 18 | | 32 | | 21 | | 44 | | 37 | | 70 | |
| **Age**, years | 58.8 (9.8) | 65.1 (10) | | 60.8 (10.4) | | 63.1 (10) | | 59.8 (10.1) | | 63.6 (9.8) | |  |
| **Sex**, % male | 63.8 | 29.5 | | 50.5 | | 36.7 | | 56.1 | | 38.6 | |  |
| **Whites,** % | 33.8 | 54.9 | | 41.2 | | 46.6 | | 34.4 | | 58.5 | |  |
| **Blacks,** % | 34.6 | 20.0 | | 23.0 | | 26.8 | | 43.0 | | 13.7 | |  |
| **Hispanics,** % | 21.0 | 19.6 | | 28.4 | | 14.8 | | 20.3 | | 17.7 | |  |
| **Chinese,** % | 10.7 | 5.5 | | 7.4 | | 11.8 | | 2.3 | | 10.2 | |  |
| **Education**, some college degree % | 61.4 | 73.0 | | 57.1 | | 75.1 | | 58.3 | | 77.5 | |  |
| **Current smoking**, % | 22.9 | 4.6 | | 19.5 | | 6.5 | | 22.1 | | 6.3 | |  |
| **Alcohol intake**, g/day | 7.6 (18) | 4.9 (10.5) | | 5 (15.8) | | 6.4 (11) | | 4.3 (13.9) | | 9 (14.3) | |  |
| **Body mass index**, kg/m2 | 28.9 (5.7) | 26.9 (4.8) | | 28.3 (5.3) | | 27.4 (5.1) | | 29.5 (5.7) | | 26.6 (4.6) | |  |
| **% Lean** | 24.8 | 38.8 | | 27.0 | | 36.0 | | 21.6 | | 39.4 | |  |
| **% Overweight** | 39.6 | 38.3 | | 41.2 | | 38.3 | | 37.3 | | 41.0 | |  |
| **% Obese** | 35.6 | 23.0 | | 31.8 | | 25.7 | | 41.1 | | 19.7 | |  |
| **Physical Activity**, MET-min/week | 2,235 (3,036) | 2,979 (3,596) | | 2,110 (2,583) | | 3,112 (3,505) | | 2,304 (3,055) | | 3,150 (3,367) | |  |
| **Dietary factors** |  |  | |  | |  | |  | |  | |  |
| **Fruit intake**, serving/day | 1.0 (1.0) | 3.4 (1.9) | | 1.1 (0.8) | | 3.3 (2.0) | | 1.2 (1.1) | | 3.1 (1.9) | |  |
| **Vegetable intake**, serving/day | 1.8 (1.2) | 3.5 (1.7) | | 1.6 (0.9) | | 3.6 (1.8) | | 1.5 (1.0) | | 3.7 (1.6) | |  |
| **Whole grain,** serving/day | 0.4 (0.5) | 1.4 (0.8) | | 0.5 (0.5) | | 1.2 (0.8) | | 0.5 (0.6) | | 1.3 (0.8) | |  |
| **Fish,** serving/day | 0.3 (0.3) | 0.3 (0.4) | | 0.2 (0.2) | | 0.5 (0.5) | | 0.2 (0.3) | | 0.4 (0.4) | |  |
| **Unprocessed Meat,** serving/day | 0.6 (0.4) | 0.3 (0.3) | | 0.5 (0.4) | | 0.3 (0.4) | | 0.5 (0.4) | | 0.3 (0.4) | |  |
| **Processed Meat,** serving/day | 0.4 (0.4) | 0.08 (0.1) | | 0.3 (0.3) | | 0.1 (0.2) | | 0.4 (0.4) | | 0.1 (0.2) | |  |
| **Refined grains,** serving/day | 1.5 (1.0) | 0.9 (0.8) | | 1.3 (0.9) | | 1.1 (0.9) | | 1.3 (1.0) | | 1.0 (0.9) | |  |
| **Soda,** serving/day | 1.0 (1.4) | 0.05 (0.3) | | 0.6 (1.2) | | 0.2 (0.7) | | 0.9 (1.4) | | 0.1 (0.4) | |  |

Table G- Multivariate-Adjusted 5-year Change in Waist Circumference according to Quintiles of Dietary Diversity of healthy and unhealthy food items by race-ethnicity in 2,505 US adults

|  | Whites | | Blacks | | | Hispanics | | | Chinese | | |
| --- | --- | --- | --- | --- | --- | --- | --- | --- | --- | --- | --- |
|  | Healthy foods | Unhealthy foods | Healthy foods | Unhealthy foods | | Healthy foods | Unhealthy foods | | Healthy foods | Unhealthy foods | |
|  | Mean change (95% CI) , cm | | Mean change (95% CI) , cm | | | Mean change (95% CI) , cm | | | Mean change (95% CI) , cm | | |
| Quintiles of diversity metrics |  | |  | | |  | | |  | | |
| Count (healthy \| unhealthy) |  | |  | | |  | | |  | | |
| 9 \| 3 | 1.5 (0.6,2.4) | 1.3 (0.3,2.4) | 1.4 (0.2,2.5) | | 2.5 (1.3,3.7) | 3.1 (2.1,4) | | 1.8 (0.6,3) | 1.1 (-0.2,2.4) | | 1.1 (0.3,1.9) |
| 14 \| 6 | 1.1 (0.3,1.9) | 1.5 (0.6,2.4) | 1.9 (0.5,3.4) | | 0.7 (-1.1,2.6) | 1.6 (0.7,2.5) | | 1.5 (0.4,2.5) | 1 (-0.1,2.2) | | 2 (0.7,3.4) |
| 17 \| 8 | 2.0 (1.2,2.9) | 1.5 (0.7,2.4) | 0.9 (-0.4,2.2) | | 1.1 (-0.1,2.3) | 0.8 (-0.5,2.1) | | 2.6 (1.5,3.6) | 3.1 (1.9,4.4) | | 2 (0.8,3.1) |
| 20 \| 10 | 1.5 (0.7,2.3) | 1.7 (0.7,2.8) | 1.8 (0.5,3.0) | | 2.1 (0.6,3.7) | 1.8 (0.4,3.2) | | 3.1 (1.4,4.8) | 1.8 (0.7,2.9) | | 2.1 (0.3,4) |
| 25 \| 14 | 1.8 (0.6,2.9) | 1.7 (0.8,2.6) | 0.9 (-0.6,2.4) | | 1.0* (-0.3,2.2) | 3.3 (1.9,4.8) | | 2.3 (0.9,3.7) | 1.2 (-0.2,2.6) | | 2.7 (0.8,4.7) |
| p-trend | 0.64 | 0.64 | 0.67 | | 0.34 | 0.69 | | 0.42 | 0.64 | | 0.13 |
| Evenness |  |  |  | |  |  | |  |  | |  |
| 0.940 \| 0.915 | 1.6 (0.7,2.5) | 1.0 (0.0,2.0) | 1.2 (-0.3,2.6) | | 1.5 (0.2,2.7) | 3.0 (1.4,4.5) | | 2.0 (0.9,3.1) | 2.5 (0.4,4.5) | | 1.0 (-0.2,2.2) |
| 0.973 \| 0.958 | 1.3 (0.5,2.0) | 2.3 (1.4,3.1) | 2.1 (0.6,3.6) | | 1.6 (0.3,2.9) | 1.9 (0.6,3.2) | | 2.4 (1.3,3.5) | 0.8 (-0.5,2.1) | | 1.9 (0.6,3.2) |
| 0.984 \| 0.974 | 2.5 (1.4,3.5) | 2.1 (1.1,3.1) | 2.1 (0.7,3.4) | | 0.9 (-0.6,2.4) | 2.4 (1.2,3.5) | | 1.8 (0.7,3.0) | 1.6 (0.6,2.6) | | 1.3 (0.1,2.5) |
| 0.991 \| 0.984 | 0.7 (-0.3,1.7) | 1.2 (0.4,2.0) | 1.4 (0.2,2.6) | | 2.6 (1.3,3.9) | 1.3 (0.4,2.2) | | 2.3 (1.2,3.3) | 2 (0.9,3.0) | | 1.6 (0.4,2.7) |
| 0.996 \| 0.993 | 1.7(0.8,2.7) | 1.1 (0.3,2.0) | 0.7(-0.5,1.9) | | 0.5 (-0.7,1.8) | 2.4 (1.4,3.4) | | 2.3 (1.0,3.7) | 1.7 (0.4,3.0) | | 2.7 (1.3,4.0) |
| p-trend | 0.77 | 0.81 | 0.84 | | 0.83 | 0.29 | | 0.76 | 0.83 | | 0.16 |
| Dissimilarity |  |  |  | |  |  | |  |  | |  |
| 0.53 \| 0.59 | 1.1 (0,2.3) | 1.0 (0.1,1.9) | 1.0 (-0.4,2.4) | | 2.0 (0.4,3.5) | 1.0 (-0.2,2.3) | | 1.1 (-0.1,2.4) | 1.2 (0.4,2.0) | | 2.3 (0.9,3.7) |
| 0.59 \| 0.65 | 2.6 (1.3,3.8) | 1.6 (0.6,2.5) | 0.9 (-0.3,2.2) | | -0.1 (-1.6,1.4) | 1.4 (0.3,2.6) | | 1.7 (0.7,2.7) | 2.2 (1.2,3.1) | | 2.0 (0.9,3.1) |
| 0.62 \| 0.68 | 1.1 (0.3,1.9) | 1.4 (0.5,2.3) | 1.4 (0.1,2.8) | | 0.9 (-0.4,2.3) | 2.2 (1.1,3.3) | | 2.5 (1.5,3.5) | 2.0 (0.2,3.8) | | 0.9 (-0.9,2.7) |
| 0.66 \| 0.71 | 1.3 (0.5,2) | 2.3 (1.5,3.0) | 1.5 (0.3,2.8) | | 2.4 (1.2,3.6) | 2.9 (1.7,4.1) | | 3.9 (2.5,5.3) | 1.1 (-0.7,2.9) | | 1.7 (0.8,2.6) |
| 0.72 \| 0.76 | 1.8 (1,2.6) | 1.6 (0.4,2.7) | 2.0 (0.6,3.4) | | 1.9 (0.8,3.0) | 2.9*(1.8,3.9) | | 1.9 (0.8,3.1) | 3.1 (0.9,5.2) | | 1.6 (0.5,2.7) |
| p-trend | 0.85 | 0.21 | 0.24 | | 0.36 | 0.01 | | 0.09 | 0.30 | | 0.39 |

Values are multivariate-adjusted mean (95%CI). MV model included age (years), sex, energy (kcal/day), race/ethnicity (White, Black, Black, Hispanic, Chinese), education (<high school, high school, some college, college graduate), field center, smoking status (never, former, current and pack-years of cigarette smoking), alcohol use (g/day), and physical activity (active and inactive leisure, MET-min/wk). *Statistically significant difference from the lower quintile (p-value < 0.05)

Table H - HRs (95% CIs) of type II diabetes for 1-interquintile rage (IQR) unit of dietary diversity of healthy and unhealthy foods in 5,160 U.S. adults

|  | Whites | Blacks | Hispanics | Chinese |
| --- | --- | --- | --- | --- |
| case/person-years | 192/22,814 | 166/11,882 | 162/10,024 | 68/6,004 |
| Healthy foods |  |  |  |  |
| Count (IQR= 16) |  |  |  |  |
| Multivariate model^1^ | 0.80 (0.53,1.19) | 1.17 (0.79,1.73) | 0.89 (0.58,1.36) | 1.09 (0.52,2.32) |
| Multivariate model^2^ | 0.95 (0.65,1.38) | 1.48 (1.04,2.11) | 1.28 (0.86,1.92) | 1.07 (0.54,2.13) |
| Evenness (IQR=0.06) |  |  |  |  |
| Multivariate model^1^ | 0.88 (0.70,1.10) | 1.28 (0.93,1.77) | 1.07 (0.79,1.46) | 1.41 (0.58,3.43) |
| Multivariate model^2^ | 0.88 (0.71,1.09) | 1.30 (0.95,1.78) | 1.21 (0.87,1.68) | 1.45 (0.58,3.61) |
| Dissimilarity (IQR=0.18) |  |  |  |  |
| Multivariate model^1^ | 1.00 (0.68,1.45) | 0.98 (0.69,1.40) | 1.09 (0.73,1.64) | 1.13 (0.59,2.19) |
| Multivariate model^2^ | 0.96 (0.66,1.40) | 0.86 (0.61,1.22) | 0.97 (0.66,1.44) | 1.06 (0.53,2.13) |
| Unhealthy foods |  |  |  |  |
| Count (IQR= 11) |  |  |  |  |
| Multivariate model^1^ | 1.50 (0.91,2.49) | 1.03 (0.67,1.58) | 0.73 (0.44,1.21) | 1.12 (0.46,2.75) |
| Multivariate model^2^ | 1.17 (0.79,1.72) | 1.23 (0.88,1.72) | 1.23 (0.81,1.87) | 1.01 (0.47,2.17) |
| Evenness (IQR=0.08) |  |  |  |  |
| Multivariate model^1^ | 1.16 (0.89,1.52) | 0.96 (0.78,1.18) | 0.95 (0.68,1.32) | 0.85 (0.58,1.24) |
| Multivariate model^2^ | 1.23 (0.92,1.63) | 1.01 (0.81,1.26) | 0.99 (0.75,1.32) | 0.79 (0.54,1.15) |
| Dissimilarity (IQR=0.17) |  |  |  |  |
| Multivariate model^1^ | 0.72 (0.53,0.98) | 0.87 (0.64,1.16) | 0.97 (0.68,1.38) | 0.83 (0.53,1.31) |
| Multivariate model^2^ | 0.70 (0.51,0.98) | 0.91 (0.68,1.21) | 0.99 (0.71,1.38) | 0.81 (0.51,1.27) |

Values are HR (95%CI). Multivariate-adjusted model 1 included age (years), sex, race/ethnicity (White, Black, Black, Hispanic, Chinese), education (<high school, high school, some college, college graduate), field center, smoking status (never, former and current smoker, and pack/years of cigarette smoking), energy (kcal/day), alcohol use (g/day), physical activity (active and inactive leisure, MET-min/wk), dietary supplement use (yes/no). Multivariate model 2 included further adjustment for BMI (kg/m2) and baseline waist circumference (cm).
